# Interaction of Deubiquitinase 2A-DUB/MYSM1 with DNA Repair and Replication Factors

**DOI:** 10.3390/ijms21113762

**Published:** 2020-05-26

**Authors:** Carsten Kroeger, Reinhild Roesler, Sebastian Wiese, Adelheid Hainzl, Martina Vanessa Gatzka

**Affiliations:** 1 Faculty of Medicine, University of Ulm, 89081 Ulm, Germany; carsten.kroeger@uniklinik-ulm.de (C.K.); adelheid.hainzl@uni-ulm.de (A.H.); 2 Mass Spectrometry Core Facility, University of Ulm, 89081 Ulm, Germanysebastian.wiese@uni-ulm.de (S.W.); 3 Department of Dermatology and Allergic Diseases; Ulm University, 89081 Ulm, Germany

**Keywords:** apoptosis, cancer, DNA damage, γH2AX, HELLS, histone deubiquitinase, homologous recombination (HR), melanoma, MYSM1, p53

## Abstract

The deubiquitination of histone H2A on lysine 119 by 2A-DUB/MYSM1, BAP1, USP16, and other enzymes is required for key cellular processes, including transcriptional activation, apoptosis, and cell cycle control, during normal hematopoiesis and tissue development, and in tumor cells. Based on our finding that MYSM1 colocalizes with γH2AX foci in human peripheral blood mononuclear cells, leukemia cells, and melanoma cells upon induction of DNA double-strand breaks with topoisomerase inhibitor etoposide, we applied a mass spectrometry-based proteomics approach to identify novel 2A-DUB/MYSM1 interaction partners in DNA-damage responses. Differential display of MYSM1 binding proteins significantly enriched after exposure of 293T cells to etoposide revealed an interacting network of proteins involved in DNA damage and replication, including factors associated with poor melanoma outcome. In the context of increased DNA-damage in a variety of cell types in Mysm1-deficient mice, in bone marrow cells upon aging and in UV-exposed Mysm1-deficient skin, our current mass spectrometry data provide additional evidence for an interaction between MYSM1 and key DNA replication and repair factors, and indicate a potential function of 2A-DUB/MYSM1 in DNA repair processes.

## 1. Introduction

According to the “access–repair–restore model”, DNA damage responses (DDR) are highly orchestrated cellular processes that depend on chromatin remodeling and histone modifications, notably histone ubiquitination [1,2,3,4,5]. Among the most cytotoxic DNA lesions are DNA double-strand breaks (DSB) occurring intrinsically in response to metabolic and replication stress, as well as during V(D)J recombination and meiosis or after extrinsic insults including ionizing radiation and anticancer chemotherapy [6,7]. Erroneous DSB repair has been linked to accelerated aging, cellular senescence, and apoptosis, as well as DNA mutations and cancer, as seen in patients with germline or somatic mutations of factors such as BRCA1/2, FANCD1 and PLK1, and in other genetic syndromes [8,9,10]. Despite promoting genetic stability and preventing tumor initiation, during melanoma progression and metastasis, high levels of DNA repair and replication factors may enable faster replication and have been associated with poor prognosis [11]. Hence, targeted intervention with the DNA repair machinery may offer new options for tumor therapy [12,13], and interactions need to be investigated.

Several histone H2A ubiquitination sites are critically involved in the initiation of DNA repair and in the fine-tuning and plasticity of repair pathway choices and outcome. After initial DSB detection and phosphorylation events mediated by checkpoint kinase ATM, E3 ubiquitin ligases, such as RNF8 and RNF168, catalyze H2A/X ubiquitination on K63 and K13-15, respectively, generating a platform for the sequential assembly and concentration of additional DNA repair proteins, like BRCA1 and 53BP1 [3,14,15,16]. Two main pathways may then alternatively be utilized for actual DSB repair, depending on the cellular context: (1) template-mediated, high-fidelity homologous recombination (HR) or (2) error-prone, nonhomologous end-joining (NHEJ) [17,18]. Moreover, noncanonical pathways like (3) single strand annealing (SSA) and (4) alternative end joining (Alt-EJ) are available [19]. Apart from protein interactions, chromatin environment, cell cycle phase, and DNA end resection repair, pathway choice may be regulated by the differential ubiquitination of H2A K13/15 and K127/129 via RNF168 and BRCA1 [17]. In parallel, mono-ubiquitination of H2A on lysine residue K119 by polycomb-repressive complex 1 (PCR1)-components BMI1/Ring1a and RNF2/Ring1b is required for efficient DSB repair to silence gene transcription around the break site [20,21]. Notably, H2A K119 mono-ubiquitination has also been implicated in nucleotide excision repair (NER) of bulky lesions in response to UV-damage in the skin, with particular relevance for skin cancer [22,23].

Fine-regulation of the ubiquitination status of H2A K63, K11-15, and K119 is achieved by various deubiquitinases (DUBs), such as USP3, USP16, USP21, BAP1, BRCC36, and others [23,24,25,26]. Each DUB may serve distinct functions in chromatin relaxation, the reversal of transcriptional silencing, and the resolve of repair complexes, and thereby influence pathway choice and outcome in DSB repair and in DNA replication stress responses. Disruption of the intricate balance of ubiquitin marks due to the loss-of-function of a DUB has been shown to severely impact DSB repair, and may promote cancer formation, as indicated by tumor suppressor functions of BAP1, USP16, and other DUBs [23,27,28,29,30].

Like tumor suppressor proteins BAP1, USP16, and USP21, the enzyme Myb-like Swirm and MPN domain containing 1 (2A-DUB/Mysm1) catalyzes the deubiquitination of histone H2A on lysine residue K119 [31]. Originally, MYSM1 was discovered as a regulator of androgen receptor-dependent gene activation in prostate cancer [31], and more recently, has also been implicated in poor outcomes of colorectal cancer [32] and in melanoma growth [33]. Analyses of Mysm1-deficient mouse models (Mysm1^tm1^) revealed a complex phenotype with abnormalities in various tissues, as reflected by reduced body weight and size, white belly spot, and kinked tail, as well as altered maintenance of hematopoietic stem cells (HSC) and defective B cell, T cell, natural killer cell and dendritic cell development, and other defects [34,35,36,37,38]. Clinical features of human patients with *MYSM1* gene defects closely resemble the murine knockout phenotype [39,40] and are summarized as bone marrow failure syndrome (BMFS) 4 [41]. Mechanistically, defective development of Mysm1-deficient mice correlated with altered gene transcription, increased apoptosis, and increased DNA damage in a variety of tissues, and was largely rescued by concomitant deletion of tumor suppressor p53 [38,42,43,44]. Although Puma was identified as critical mediator of p53 transcriptional stress responses, increased apoptosis, and DNA-damage in Mysm1^−/−^ multipotent progenitors (MPP) and other cell types [45], defective lymphocyte development was not rescued by simultaneous Puma-ablation. Hence, Mysm1 may affect stress responses as well as p53 activity at distinct target promoters in a cell type- and chromatin context-dependent manner, warranting further investigation.

To follow up on our observations of increased spontaneous and induced DNA damage in various cell types of Mysm1-deficient mice, and of altered MYSM1 expression in tumor cells, we used a mass spectrometry-based proteomics approach to explore MYSM1 functions in DNA damage responses. The identification of several novel candidate interacting partners place MYSM1 in a functional network related to different DNA repair pathways and DNA replication, with potential relevance for normal development and tumorigenesis.

## 2. Results

### 2.1. MYSM1 Is Recruited to γH2AX Foci upon Chemical Induction of DNA Damage with Etoposide in Human Peripheral Blood Mononuclear Cells (PBMC) and Tumor Cell Lines

In initial protein expression and localization analyses, significant amounts of MYSM1 were detectable in a variety of human cell types, including PBMC, KG-1a myeloid leukemia cells, A375 and SK-MEL-28 melanoma cells, HepG2 liver cancer cells, and A172 glioblastoma cells, mainly in the nuclei (Figure S1A–F). Correspondingly, on the mRNA level, MYSM1 was expressed in human PBMC and in lymphoma and leukemia cell lines to variable extents (Figure S1G).

**Figure 1:**
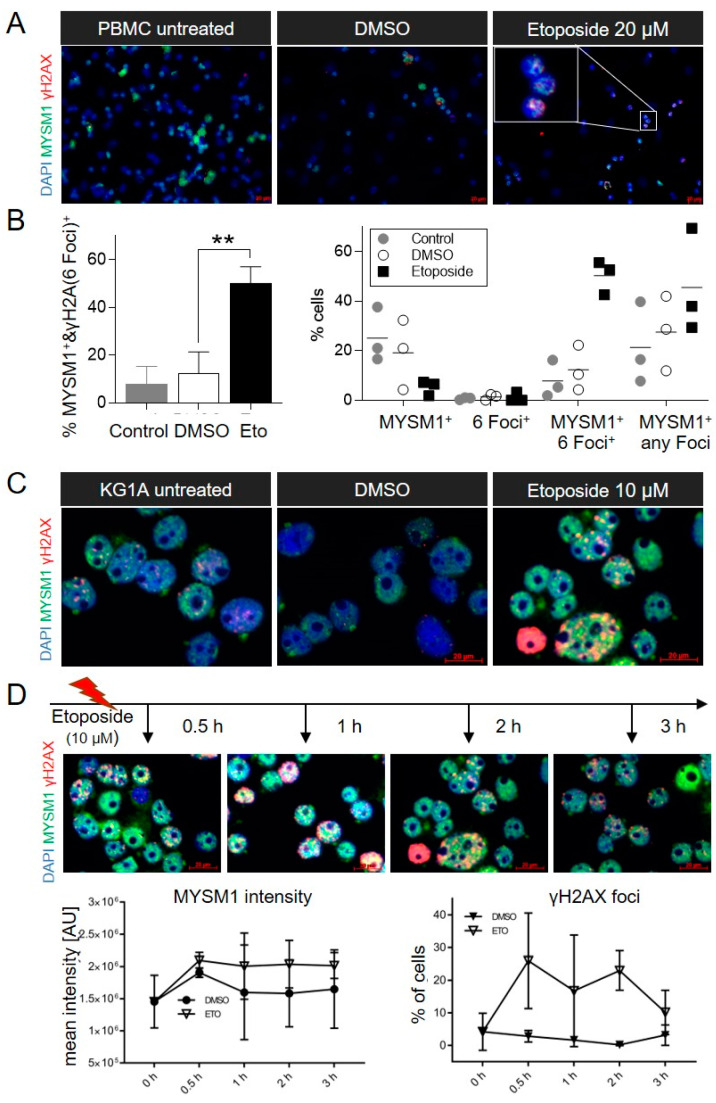
MYSM1 colocalizes with γH2AX foci upon DNA damage induction. (**A**). Immunofluorescent (IF) analyses of MYSM1 and γH2AX in human PBMC treated with etoposide (20 µM) or DMSO for 16 h compared with untreated PBMC (original magnification 40X). In all IF images shown in Figure 1: MYSM1 green, γH2AX foci red, nuclei blue, double-positive (DP) foci yellow. Representative images of at least three independent experiments were chosen. (**B**). Quantification of colocalization of MYSM1 with γH2AX foci in PBMC under the conditions indicated. At least 15 high power fields per sample in 3 independent experiments were counted. (**C**). IF analyses of MYSM1 and γH2AX in KG-1a myeloid leukemia cells upon etoposide (10 μM) exposure for 2 h compared with DMSO (original magnification 62,5X). (**D**). Time-course of MYSM1 intensity and γH2AX foci formation after exposure of KG-1a cells to etoposide (10 μM) for indicated time periods.

Based on detected increases in a marker of DNA-damage, phosphorylated histone 2AX (γH2AX), in different tissues of Mysm1-deficient mice and in UV-exposed Mysm1^−/−^ skin [33,38], we hypothesized that 2A-DUB/Mysm1 may have a direct role in DNA damage repair. In an in vitro model of chemical DSB DNA damage induction with etoposide (Eto, 20 μm) in human PBMCs for 16 h, the number of γH2AX foci was substantially increased compared with DMSO-treated and untreated control PBMC in immunofluorescent (IF) analyses (Figure 1A). Importantly, MYSM1 redistributed upon etoposide treatment and colocalized with γH2AX foci in the nuclei of PBMC (Figure 1A, insert, and Figure S1H), which is indicative of a potential interaction with the DNA repair machinery. Accordingly, the overall number of PBMC with nuclear foci double-positive for MYSM1 and γH2AX was significantly increased upon etoposide exposure relative to the controls in the corresponding quantifications (Figure 1B).

Similarly, increased numbers of γH2AX foci, the redistribution of MYSM1, and the colocalization of MYSM1 with γH2AX foci occurred in KG-1a leukemia cells treated with 10 μM etoposide for 2 h (Figure 1 C). Proper DNA damage induction and the initiation of DNA repair responses upon etoposide in the samples was verified by recruitment of DDR factor 53BP1 (Figure S2). Nuclear MYSM1 intensity increased as early as 0.5 h post etoposide exposure of KG-1a cells, and stayed elevated for 1–3 h in parallel with increases in γH2AX^+^ cells as indicated in the quantifications (Figure 1D). In A375 melanoma cells that appeared highly sensitive to etoposide, similar increases in MYSM1 and γH2AX were found (Figure S3).

### 2.2. Identification of MYSM1 Interaction Partners by Co-Immunoprecipitation-coupled Mass Spectrometry in MYSM1-Flag 293T Cells

**Figure 2:**
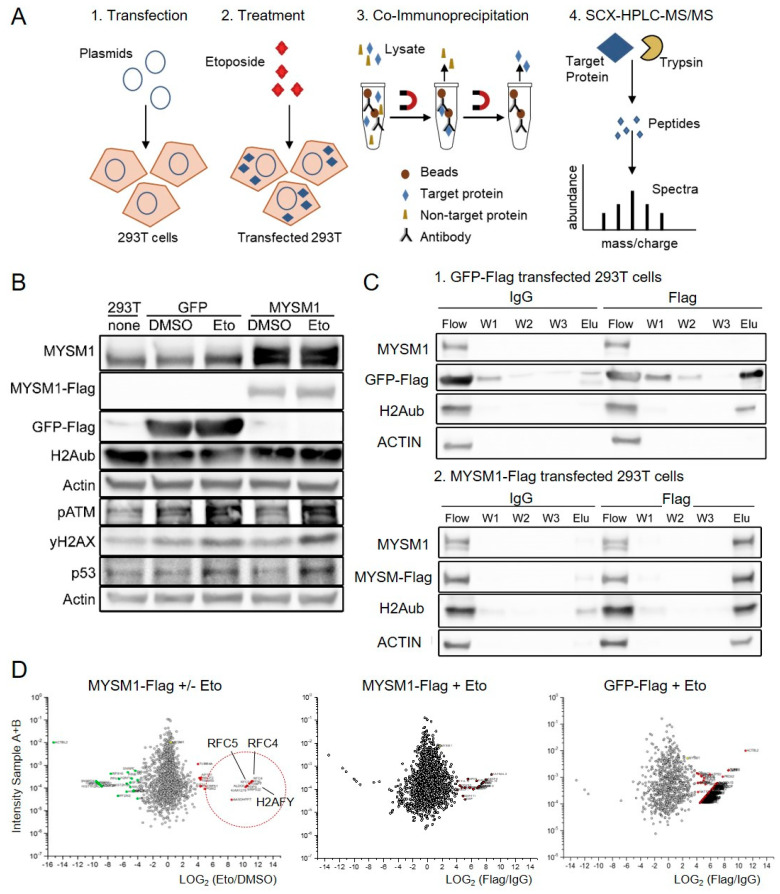
Analysis of protein interactions of MYSM1 in response to etoposide in MYSM1-Flag 293 T cells. (**A**). Workflow of the proteomics approach with 1. Overexpression, 2. Treatment, 3. Co-Immunoprecipitation (IP), 4. SCX-HPLC Mass spectrometry. (**B**). Western blot (WB) analysis of MYSM1, H2Aub, and DDR factors in 293T cells transiently transfected with either GFP-Flag or MYSM1-Flag upon treatment with etoposide (Eto, 10 μM) or DMSO for 6 h as indicated. MYSM1-Flag and endogenous MYSM1 migrated at slightly different speed in SDS-PAGE. (**C**). Detection of MYSM1, GFP-Flag, and the MYSM1 substrate H2Aub in the input and in the eluate (Elu) as well as wash fractions (W1-3) after IP with an anti-Flag antibody compared with control IgG in 293Tcells transfected with either GFP-Flag (upper) or MYSM1-Flag (lower) by WB. (**D)**. Differential display of significant MYSM1 protein interactions identified in three indicated MS sample pairs: Protein abundances calculated from the total label-free quantification (LFQ) intensity of both samples compared are plotted against the ratio of LFQ intensity (log2) between specific samples and control as indicated. Proteins in the dotted circle are enriched above the cut off value set at 2-4 fold.

To identify new candidate MYSM1 interaction partners and further investigate the molecular function of MYSM1 in DNA damage responses (DDR), we subsequently performed co-immunoprecipitation (co-IP) experiments in 293T cells transiently overexpressing MYSM1-Flag or GFP-Flag upon treatment with etoposide, followed by an SCX-HPLC MS/MS mass spectrometry proteomics approach [46,47]. The work flow of our proteomics approach is summarized in Figure 2A: (1) Overexpression: Due to the relatively difficult detectability of endogenous MYSM1 in the lysates of the cell lines analyzed (not shown) and the lack of validated anti-MYSM1 antibodies for IP, flag-tagged MYSM1 was transiently overexpressed via pcDNA3.1(+) vector in 293T cells for 48 h as a “bait” protein for co-IP. 293T cells transfected with GFP-Flag were used as controls; (2) Treatment: To induce DSB, cells were treated with 10 µM etoposide (Eto) during the last 6 h of transfection, using DMSO as a negative control; (3) Co-IP: Prior to preparation of whole cell extracts with RIPA-buffer and Western blot or co-IP with either anti-Flag or control IgG. Eluates from all 8 co-IP conditions (Table S1) were fractionated by SDS-PAGE, digested, and subjected to (4) SCX-HPLC-MS/MS mass spectrometry with an Orbitrap Velo Pro, as described in the Methods section.

At first, robust expression of GFP-Flag and MYSM1-Flag fusion proteins, as well as DNA damage induction by etoposide, were confirmed in comparison to endogenous MYSM1 levels in parental and DMSO-treated 293 T cells by Western blot (Figure 2B) and by IF analyses (not shown). Etoposide-induced DNA damage responses at the time of analysis were verified by increased levels of pATM, γH2AX, and p53 compared with DMSO controls (Figure 2B). As an internal quality control, successful and specific co-IP of fusion proteins by anti-Flag compared with control IgG were affirmed in lysates from MYSM1-Flag transfected 293T cells vs. GFP-Flag controls, respectively, prior to sample analysis by mass spectrometry (Figure 2C). In addition, as proof of principle of the co-IP approach, MYSM1 interaction with ubiquitinated histone H2A (H2Aub), a known substrate of the H2A deubiquitinase, was detectable in the anti-Flag pull-down from MYSM1-Flag transfectants, as expected (Figure 2C, lower panel). The small amounts of Actin found in these samples may account for potential interactions of MYSM1 with components of the cytoskeleton, such as Actin and also Lamin B, which were previously shown to facilitate chromatin positioning, complex assembly, and recruitment of chromatin remodelers SMARCA5/SNF2H [17,48], HELLS/SMARCA6 [10], and others.

Subsequently, the IP eluates from MYSM1-Flag and GFP-Flag transfected 293T cells, processed according to the workflow in Figure 2A, were subjected to SCX-HPLC MS/MS mass spectrometry in order to identify novel MYSM1 interactions in DDR. In both sample groups, samples were treated either with etoposide (Eto) or DMSO, and IP was performed with anti-Flag or control IgG, respectively, resulting in a total of eight mass spec samples (Table S1 and Figure S5A). The raw MS data were analyzed using the MaxQuant Software (Martinsried, Germany). In total, 2247 distinct interacting proteins, represented by at least one unique peptide, were retrieved in all eight sample conditions. Differential protein abundances were calculated by plotting the combined intensities of two corresponding samples against the ratio of the intensity between specific sample and control (Figure 2D).

Specific potential MYSM1 interactors were classified as proteins with at least > 16-fold (log2(16) = 4) differential enrichment in MYSM1-Flag anti-Flag vs. IgG eluates, but not in the GFP-Flag eluates. With this cut-off, a total of 178 candidate MYSM1 interaction partners were identified (Figure S4B). After removal of common contaminants, such as keratins, histones, tubulins, actins, and others, in accordance with 71 reported “CRAPome” control experiments (crapome. org, [49]), the number of potential specific MYSM1 interactors was reduced to 112. As an internal validation, MYSM1 itself was highly enriched (log2(1024) = 10) in MYSM1-flag vs. GFP-flag transfected samples and in anti-Flag vs. IgG co-IP. Based on highest differential enrichment in etoposide-treated MYSM1-flag eluates vs. corresponding DMSO controls, DSB response-specific candidate interacting proteins were ordered and subsequently structured in functional groups by pathway analyses (Table S2). Pathway analyses of MYSM1 candidate interaction partners revealed functions in cellular metabolism, oxidative phosphorylation, apoptosis, drug metabolism, and DNA repair (Figure S4C).

### 2.3. Mechanistic Analyses of MYSM1 Interactions Partners Differentially Enriched in Etoposide Treated 293T Cells Reveals a Network of DNA-damage and Replication Factors

**Figure 3:**
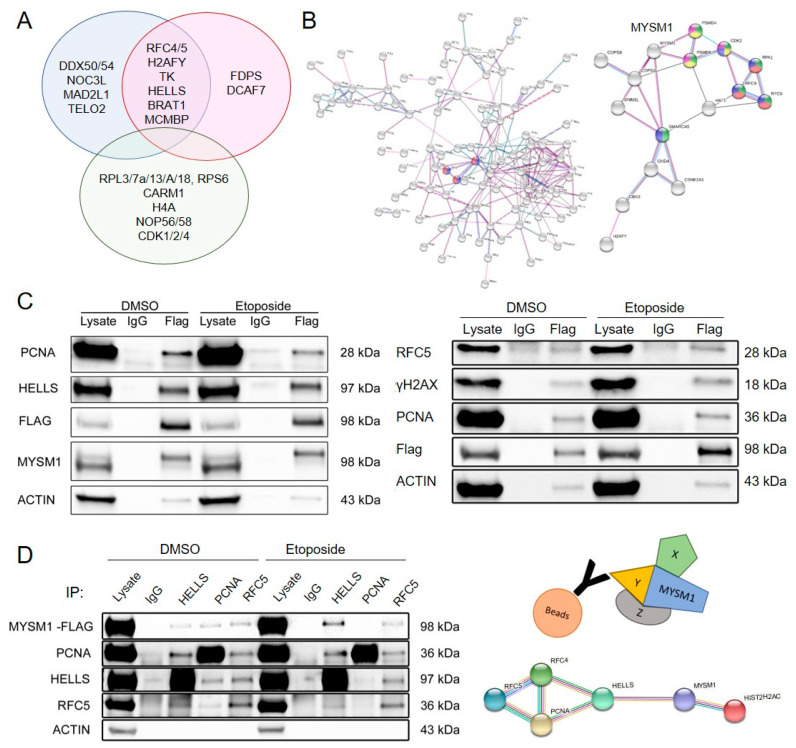
Identification of MYSM1 interaction partners in DNA-damage responses by SCX-HPLC-MS/MS mass spectrometry and verification by direct and reverse Co-IP. (**A**). Differential display of top candidate proteins significantly enriched in the specific MYSM1-Flag Eto/DMSO sample group (red) compared with MYSM1-Flag Eto Flag/IgG (blue) and GFP-Flag Eto Flag/IgG control groups (green). (**B**). String network analysis of overall MYSM1 protein interactions identified by MS (left panel) and specific interactions in DNA damage response pathways (right panel). (**C**). WB confirmation of specific MYSM1 interactions with RFC5, PCNA, and HELLS in vitro by co-IP with an anti-Flag Ab vs. control IgG followed by WB with indicated Ab in MYSM1-Flag transfected 293T cells treated with etoposide and in DMSO controls. (**D**). Reverse co-immunoprecipitation of MYSM1 with antibodies against HELLS, PCNA, and RFC5 vs. control IgG in lysates from MYSM1-Flag transfected 293 T cells treated with etoposide or DMSO for 6 h (left panel). The graphic on the right shows the potential pull-down of proteins complexed with MYSM1 and the String network.

In differential display analyses, relevant DNA damage-dependent MYSM1 candidate interaction partners were defined as proteins differentially enriched by at least 4-fold (log2(4) = 2) in MYSM1-Flag eluates upon etoposide vs. DMSO treatment by anti-Flag vs. IgG IP, but not present in GFP-Flag eluates (Figure 3A). As top candidate MYSM1 interaction partners with the highest specific enrichment upon etoposide treatment, we identified two subunits of the clamp loader complex replication factor C (RFC) 4 and RFC5, macroH2A.1, as well as thymidine kinase 1 (TK1) (Figure 2D or Figure 3A). In addition, with slightly lower stringency, the DNA helicase HELLS, minichromosome maintenance complex binding protein (MCMBP), and BRCA1-associated ATM activator 1 (BRAT1) were found to bind to MYSM1 upon DNA damage induction. Furthermore, mild interactions of MYSM1 with POLD1 and MSH6 and with Lamin B1, involved in higher-order chromatin structure and anchoring of chromatin, were detectable. In a differential display of the mass spec hits enriched under conditions of DNA damage in etoposide/DMSO (blue) and etoposide anti-Flag/IgG (red) samples, the specific DNA-damage-dependent MYSM1 interactors are revealed in the intersection (purple) compared with noninteractors upregulated in etoposide-treated GFP-Flag transfectants (green) (Figure 3A).

Based on the list of specific hits, STRING analyses of newly-identified, known, and predicted protein–protein-interactions of MYSM1 and its binding partners revealed a network of DNA repair and replication factors with abundant functional relations between most members identified (http://string-db.org) [50]. Additionally, the STRING network analyses placed PCNA in a functional node with RFC4, RFC5, and MYSM1, as well as other proteins involved in DNA replication and repair (Figure 3B). Moreover, the mass spec results revealed increased potential interactions of MYSM1 with metabolic enzymes, mitochondrial proteins, and components of the cytoskeleton during DNA repair (complete list in the suppl. data). However, under the conditions used here, no direct interaction with either p/CAF, p53 or TRAF3 and TRAF6—i.e., factors previously shown to bind to MYSM1 in prostate cancer cells or immune cells, respectively, [31,45,51]—were detected.

Subsequently, the specificity of the interaction of the three top candidate MYSM1 interacting proteins identified by mass spec with high enrichment upon DSB induction namely, RFC4, RFC5, and HELLS, as well as the putative interactor PCNA, was confirmed by direct and reverse Co-IP; 293T cell lysates were prepared as described and subjected to co-IP with anti-Flag and control IgG followed by Western Blot with specific antibodies against PCNA, HELLS, and RFC5 in two independent experiments (Figure 3C). The validation co-IPs again retrieved the known MYSM1 interaction partner, H2A, as detected with an antibody against γH2AX. Proper induction of DNA-damage and increased amounts of γHAX2, 53BP, and RAD51 after etoposide exposure were confirmed by Western Blot, showing some DNA-damage in the DMSO-treated samples as well (Figure S5). Moreover, in a reverse co-IP approach, significant amounts of MYSM1 were recovered with specific antibodies against HELLS and RFC5, as well as PCNA, and enriched upon etoposide exposure, as detected by an anti-Flag antibody (Figure 3D). Importantly, co-IP with anti-HELLS and anti-RFC5 pulled down PCNA, RFC5, and HELLS, respectively, confirming the potential interactions between the candidate proteins and MYSM1 in a larger protein complex.

### 2.4. Evaluation of the Role of 2A-DUB/Mysm1 in DNA Damage Responses and Replication in Murine HSPC upon Aging and in Human Melanoma Cells

**Figure 4:**
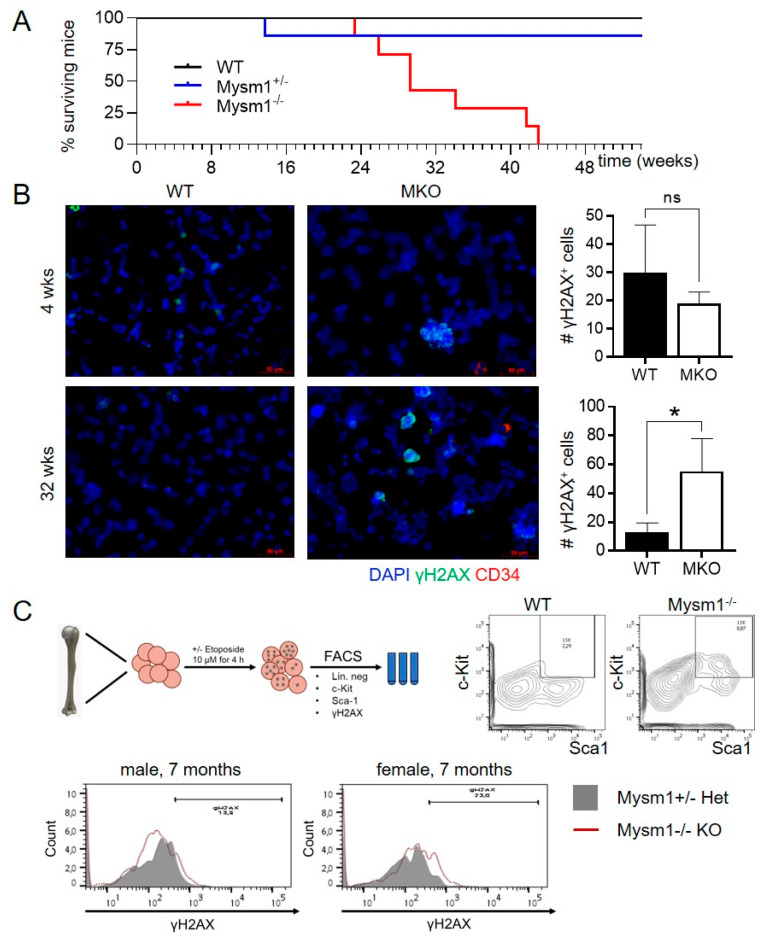
Lethality and DNA damage in aging Mysm1-deficient mice. (**A**). Survival curve of Mysm1^tm1a^ mice compared with controls upon aging for 24 months. (**B**). IF analysis of γH2AX foci in bone marrow cells of 7–8-month-old Mysm1^tm1a^ *vs*. age-matched control mice (γH2AX green, CD34 red, nuclei blue, 40X) and corresponding quantifications. (**C**). FACS analysis of γH2AX in sorted BM HPSCs from aged Mysm1^tm1a^ (dotted red line) *vs*. wild-type control mice (grey) (representative mice shown). Positive controls were treated with etoposide 10 μM for 4 h (not shown).

In a systematic survival analysis, an average maximal life span of 6–8 months of Mysm1^tm1a^ (Mysm1^−/−^, MKO) mice was observed, compared to up to 24 months for wild-type littermates (Figure 4A). The premature lethality of Mysm1^−/−^ mice correlated with a mild aggravation of changes in blood and immune cell composition in bone marrow (BM), blood, and secondary lymphoid organs of 7-month-old Mysm1^−/−^ compared with Mysm1^+/−^ mice, potentially reflecting successive bone marrow dysfunction (Figure S6). Indicative of a potential contribution of increased DNA-damage or replication stress in the blood cell changes, significantly increased fractions of γH2AX-positive cells were detectable in total BM from aged Mysm1^−/−^ mice compared with age-matched Mysm1^+/−^controls in IF analyses, while differences were not significant in younger mice (Figure 4B). However, due to relatively small fractions of CD34-positive cells, our quantification did not allow any precise conclusions to be made regarding the extent to which HSCs were affected.

In corresponding FACS analyses of BM cells from aged Mysm1^−/−^ mice, the overall fraction of Lin^-^Sca-1^+^c-Kit^+^ (LSK) cells, containing the HSC, was consistently expanded more than 3-fold compared with age-matched Mysm1^+/−^ controls (Figure 4C, top), potentially resulting from disturbed lineage specification and/or the expansion of the HSC pool. Within the Mysm1^−/−^ LSK-cell fraction, overall γH2AX levels were increased compared with controls (Figure 4C, bottom). However, the detection of overall increases in γH2AX levels in Mysm1^−/−^ BM cells provides only insufficient evidence for a direct impact of MYSM1 on the DNA repair capacity, because DSB may occur secondary to p53-Puma-mediated apoptosis [45]. In parallel IF analyses of cycling A375 melanoma cells, MYSM1 was found to redistribute to the nuclear periphery during M-phase of the cell cycle, and did not colocalize with either Ki-67 or condensed HH3 (Figure S7).

## 3. Discussion

To further explore the correlation between increased DNA damage and developmental defects observed in humans with genetic alterations of 2A-DUB/MYSM1 and in mouse models, we analyzed protein interactions of MYSM1 upon chemical induction of DSB DNA-damage in more detail. In summary, our current results support a role of MYSM1 in DDR: (1) MYSM1 was recruited to DNA lesions induced by etoposide in vitro in human PBMC, KG-1a myeloid leukemia cells, and in melanoma cells. (2) In mass spectrometry analyses, known DNA repair and replication factors, including helicase HELLS and RFC4/5, were identified as novel candidate interaction partners of MYSM1 upon DNA damage induction in 293T cells. (3) The specificity of the interactions of MYSM1 with RFC5, HELLS, and PCNA could be verified by direct and reverse co-IP, and (4) correlated with accumulation of γH2AX in HSPC from aged Mysm1-deficient mice.

In accordance with our hypothesis, the newly-identified top candidate MYSM1 interactors in response to etoposide, RFC4/5 and HELLS, as well as PCNA, have all been previously implicated in the maintenance of genome integrity, and carry out cell cycle-dependent dual functions in DNA replication and DNA repair [52,53,54,55]. However, although the identification of RFCs suggested a potential role of MYSM1 in DNA replication or mismatch repair, interactions with PCNA, DNA polymerases POLA, D, and E, or mismatch repair proteins (MMR) were below the cutoff in our screening, and only mild interactions with POLD1 and MSH6 were uncovered. Chromatin remodeling kinetics, cell cycle dynamics, and other experimental factors may have influenced the composition of protein–protein complexes in our approach. Therefore, individual co-IP and functional interactions analyses need to be performed for verification, as shown here for PCNA. In addition, posttranslational modifications of MYSM1, such as phosphorylation or ubiquitination, may sequentially occur in context of DNA damage and regulate alternative interaction options and functions, which is comparable with PCNA ubiquitination or BAP phosphorylation in UV-induced DNA-damage responses [27,56]. Accordingly, MYSM1 was previously identified as an ATM phosphorylation substrate in DDR [57], and several potential phosphorylation sites were found in the MYSM1 protein of still unclear functional relevance.

Molecularly, both processes, i.e., DNA replication and repair, rely on an interplay of chromatin remodeling, DNA processing, and DNA synthesis. Moreover, significant cross-talk between DSB and non-DSB DNA repair pathways and the utilization of similar quality control mechanisms that require protein-chromatin interactions—for instance, during DNA mismatch repair (MMR), translesion synthesis (TLS), and postreplication repair (PRP)—appear to occur [58,59]. During regular cell cycle, PCNA functions as part of the normal DNA replication machinery (“replisome”) as DNA sliding clamp and processivity cofactor for Pol δ and ε in cooperation with clamp loader RFC complex possessing DNA-dependent ATPase activity [52]. In parallel, upon ubiquitination, and potentially other modifications, PNCA and RFC are involved in several DDR pathways requiring Pol δ/ε-mediated DNA synthesis, including TLS and NER, and may recruit context-specific repair factors [60]. During HR, collaboration among PCNA, RFC4/5, and Pol δ was shown to be critical for so-called postinvasion DNA synthesis, stimulating Pol δ to displace the DNA strand and to extend the strand for longer distances (>100 nt) [61]. Hence, interplay between PCNA and RFC with additional repair proteins and chromatin remodelers, such as HELLS and MYSM1, may be required in a context-dependent manner to allow for chromatin relaxation and/or up- and unloading of protein complexes.

Similarly, the lymphoid-specific DNA helicase HELLS (also Lsh, PASG, SMARCA6), a SNF2-like chromatin remodeling ATPase, has previously been shown to have various functions in DSB DNA repair, in particular in HR in heterochromatic regions in G2 [62,63], as well as in NER, transcriptional regulation, and in cell cycle control [10,55]. Decreases in HELLS correlate with genome instability. Moreover, humans with deficiency in HELLS display life-threatening immunodeficiency, centromeric instability, and facial anomalies (ICF) syndrome [55] reminiscent of the phenotype of Lsh-deficient mice [64] that share certain traits with Mysm1-deficient mice. The interaction of PCNA and RFC subunits in larger protein complexes—that may contain MCM helicase components, also identified in our current MS analysis, and other chromatin modifying enzymes—has also been implicated in the prevention of replication fork stalling and secondary DNA damage accruing in aging stem cells and under stress conditions [65,66]. However, while PCNA and RFC4 were found in proximity to stalled replication forks at nascent DNA ends in a recent proteomics screen, helicase HELLS was more abundant in the chromatin fraction more distant from the forks [67]. Interestingly, our current proteomics data now place MYSM1 in a functional network with PCNA, RFC, HELLS, and MCMBP with potential relevance in hematopoiesis and in cancer progression. However, in melanoma cells, MYSM1 was not found in proximity to either HH3 or Ki-67 in heterochromatin during cell cycle [68], warranting more detailed analyses of the putative function of the MYSM1-HELLS interaction in DDR and gene transcription.

The accumulation of DNA damage, replication stress, and upregulation of p53 contribute to HSC aging and dysfunction [69,70]. Apart from DNA repair factors and p53, modifiers of H2A mono-ubiquitination, such as PCR1 components or 2A-DUB/Mysm1, have been implicated in proper HSC maintenance and self-renewal capacity [21,34,35]. DNA repair pathway decisions in healthy HSC, with functional balance between quiescence and self-renewal, favor the use of NHEJ in quiescent cells (G0), whereas HR is activated in cycling cells (G2-M) [10,71,72]. In this context, p53 expression is required for HSC quiescence, as well as the efficient formation of 53BP1 foci, and p53 loss-of-function steers DNA repair from NHEJ to HR [16,73]. In conjunction with altered gene transcription and the induction of p53-mediated stress responses, the loss of specific functions of 2A-DUB/Mysm1 in DNA repair pathway, as per our current proteomics data, may thus contribute to defective HSC maintenance and lymphoid specification, explaining an overall selective advantage and increased fitness of p53-deficient HSC in the context of Mysm1-deficiency [38,42,45].

Like 2A-DUB/MYSM1, the newly-identified MYSM1 interaction partners, HELLS and RFC4/5, have also been implicated in tumorigenesis, specifically in melanoma growth [33]; HELLS is frequently misregulated in a variety of cancers. In human melanoma, elevated levels of HELLS were associated with tumor progression [74], and HELLS has been identified as a valid biomarker in liquid biopsies with superior prognostic value compared with LDH [75]. Similarly, RFC4 and RCF5 are involved in the G2/M checkpoint in tumors, and RFC5 expression correlates with melanoma progression [76] and may affect responses to chemotherapy [77]. In general, increased levels of DNA repair factors have been shown to facilitate tumor progression and metastasis, and were often associated with poor prognosis [11,76]. Therefore, the potential role of the MYSM1-RFC-HELLS interaction in DNA repair and genome integrity will now need to be verified in tumor samples at different tumor stages and in response to treatment. In the future, better understanding of the functions of MYSM1 and its newly-identified interaction partners in DDR may lead to the identification of additional druggable targets sensitizing certain tumor cells towards DNA-damaging agents or radiation, as per the concept of synthetic lethality [78,79].

## 4. Materials and Methods

### 4.1. Cell Culture Methods

All 293T cells, KG-1a myeloid leukemia cells, and A375 melanoma cells were purchased from the ATCC, and maintained in either DMEM or RPMI medium supplemented with 10% FBS, 1% L-Glutamine and 1% Penicilin/Streptamycin as described previously [33].

### 4.2. Expression Plasmids and Transfections

MYSM1-Flag and GFP-Flag expressing pcDNA3.1(+) vectors were purchased from Genescript (Leiden, The Netherlands), amplified, and purified prior to transfection into 293T cells using TransIT®-LT 1 (Mirus, Madison, WI, USA) according to the manufacturers’ instructions. GFP-Flag and MYSM1-Flag fusion proteins were expressed in 293T cells for 48 h with addition of 20 μM Etoposide or equivalent volume of DMSO for the last 6 h. Subsequently, cells were washed with PBS, harvested in 300 μL RIPA buffer supplemented with protein inhibitor cocktail (PIC; Sigma-Aldrich, Taufkirchen, Germany) and PMSF for lysate preparation.

### 4.3. Immunofluorescence Microscopy

IF analysis of tissue culture cells was performed as previously described [33,38]. Briefly, cells were seeded on poly-L-lysine coated slides and subsequently fixed, permeabilized and stained with specific primary antibodies against MYSM1 (HPA054291; Atlas Antibodies, Bromma, Sweden), Flag (F1804; Sigma-Aldrich, St. Louis, MO, USA), γH2A.X (phosphoS139, ab2893; Abcam, Cambridge, MA, USA), and other antibodies as indicated followed by fluorochrome-labeled species-specific secondary antibodies (A11034, A21429, A21244, A21434; Thermo Fisher, Schwerte, Germany). Nuclei were visualized by DAPI staining. Isotype IgG served as negative control in all experiments. All images were taken on Axio Imager M1 or LSM 710 systems by Zeiss (Jena, Germany). Unless indicated otherwise, the original magnification was 40×, and representative slides of at least three independent experiments are shown.

### 4.4. Co-Immunoprecipitation and Western Blot

Co-IP was performed with Protein G-coupled Dynabeads^®^ (Thermo Fisher, Waltham, MA, USA) according to manufacturer’s instructions. Briefly, 30 μl beads were incubated with 3 μg anti-Flag antibody (F1804; Sigma-Aldrich) for 10 min in PBS-T (0.2% Tween-20) at room temperature. Antibody-coupled beads were incubated overnight with 200 µg protein lysate in PBS-T at 4 °C. After washing beads in PBS-T, protein complexes were eluted in SDS loading buffer for 5 min at 70 °C. Subsequently SDS-PAGE with pre-cast 4–20% gradient gels (Bio-Rad, Feldkirchen, Germany) and Western Blotting were performed as previously described [33]. Proteins were detected with primary antibodies against Flag (F1804; Sigma-Aldrich), MYSM1 (orb137033, Biorbyt; St Louis, MO, USA), H2Aub (8240; Cell Signaling, Leiden, The Netherlands), H2AX (Abcam; ab2893, Cambridge, UK), Actin (sc-1615HRP; Santa Cruz, Heidelberg, Germany), and other specific antibodies as indicated followed by Peroxidase-coupled specifies-specific secondary antibodies (711-035-152, 715-035-150; Jackson ImmunoResearch, West Grove, PA, USA).

### 4.5. Mass Spectrometry

Co-IP protein samples were fractionated by SDS-PAGE as described above, and complete sample gel lanes were cut into 15 fragments, purified, and digested by trypsin. Peptides were further fractionated in a strong cation exchange HPLC and analyzed by mass spectrometry (SCX-HPLC-MS/MS) using electrospray ionization and CID fragmentation in a Thermo Scientific Orbitrap Velos Pro (workflow in Figure 2A).

### 4.6. Mice

Mysm1^tm1a(Komp)Wtsi^ mutant mice (Mysm1^−/−^, MKO) have been described before [33,38], and were handled according to the guidelines for animal experimentation approved by the Regierungspraesidium Tübingen, Germany (TVA 1261, 06/2016).

### 4.7. Flow Cytometry and Blood Analysis

Murine submandibular blood was captured and analyzed with a HEMAVET 950FS (Drew Scientific, Miami Lakes, FL, USA). Total murine bone marrow cells were prepared from femurs and tibias of 7-month-old Mysm1-deficient and age-matched wild-type mice as previously described. After red blood cell lysis, blood and bone marrow cells were resuspended in PBS with 2% heat-inactivated FBS, stained, and analyzed by Flow cytometry using a FACS Canto System and Flow Diva Software as described earlier [38]. Antibodies against the following markers were used for blood analyses: B220 (552771; BD Bioscience, Heidelberg, Germany), Gr-1 (12-5931-82; eBioscience, San Diego, CA), CD4 (25-0041-82, eBioscience), CD8 (48-0081-82; eBioscience), and F4/80 (MF48020, Thermo Fisher). LSK-cells were stained to determine intracellular γH2AX using antibodies against H2AX (560445; BD), Sca-1 (45-5981-80; eBioscience), c-Kit (17-1171-82; eBioscience), and mouse lineage panel (488-7772-72; Thermo Fisher).

### 4.8. Statistics

Unless indicated otherwise, p-values were calculated using a Students’s *t*-test or one-way ANOVA (analysis of variance); significance levels were denoted as follows: * = *p* < 0.05, ** = *p* < 0.01, *** = *p* < 0.005.

## 5. Conclusions

Our proteomics analysis provides additional evidence for a function of 2A-DUB/MYSM1 in DNA repair processes in collaboration with known DNA repair and replication factors, and potentially in protection from replication stress. We propose that MYSM1 interactions in DNA repair—in combination with MYSM1’s transcriptional activator and cofactor functions—may contribute to regular development and homeostasis, and may play distinct roles in tumorigenesis.

## Supplementary Materials

The following are available online at https://www.mdpi.com/1422-0067/21/11/3762/s1, Figure S1: Expression of MYSM1 in human PBMC and indicated tumor cells lines. Figure S2: Induction of DNA damage responses upon etoposide treatment in KG-1a leukemia cells Figure S3: Recruitment of MYSM1 to γH2AX foci upon DSB induction with etoposide in A375 melanoma cells. Figure S4: Procedure of the mass spectrometry analysis of eluates from Flag-IP samples Figure S5: Western blot analysis of DNA damage marker expression in 293T cells upon exposure to etoposide. Figure S6: Blood cell counts and subsets in young vs. aged Mysm1-deficient mice and their Mysm1^+/-^ littermates. Figure S7: Cell-cycle dependent distribution of MYSM1 in A375 melanoma cells. Table S1: Experimental conditions and sample groups for the mass spec proteomics approach. Table S2: Potential interaction partners of MYSM1 identified by mass spectrometry in 293T cells upon DNA damage induction by etoposide.

## Abbreviations

DDR	DNA damage response
DSB	DNA double strand break
HR	homologous recombination
