# Supplementary material for: Interaction of Deubiquitinase 2A-DUB/MYSM1 with DNA Repair and Replication Factors
Source: Int J Mol Sci. 2020 May 26;21(11):3762. doi: 10.3390/ijms21113762 (PMC7312997; doi:10.3390/ijms21113762)
Supplement: Supplementary file 1 [file ijms-21-03762-s001.pdf]

International Journal of
Molecular Sciences

MDPI

Supplementary Tables

Table S1.

#	Cell line / Bait	Condition	IP
	GFP-Flag 293T cells		
1	GFP-Flag	Etoposide [10 µM]	anti-Flag
2	GFP-Flag	Etoposide	IgG
3	GFP-Flag	DMSO	anti-Flag
4	GFP-Flag	DMSO	IgG
	MYSM1-Flag 293T cells		
5	MYSM1-Flag	Etoposide [10 µM]	anti-Flag
6	MYSM1-Flag	Etoposide	IgG
7	MYSM1-Flag	DMSO	anti-Flag
8	MYSM1-Flag	DMSO	IgG

Table S1: **Experimental conditions and sample groups for the mass spec proteomics approach**. The experimental conditions for each sample are described in the Results section and the Materials and Methods section.

Table S2

Rank	Protein Name	Protein Accession	Molecular Weight [kDa]	Abundance [log2]
	A) MYSM1 interacting proteins in DMSO treated cells (top candidates)			
1	ACTBL2	Q562R1.2	42.0	11.19
2	UBTF	P17480.1	87.4	8.72
3	IPO4	Q8TEX9.2	118.7	7.06
4	PPIB	P23284.2	23.7	6.79
5	HSP90AB2P	Q58FF8.2	44.3	6.67
	B) MYSM1 interacting proteins in etoposide treated cells (top candidates)			
1	RFC4	P35249.2	36.9	11.26
2	RFC5	P40937.1	38.5	11.12
3	H2AFY	O75367.4	39.2	10.88
4	TK1	P04183.2	25.5	3.76
5	BRAT1	Q6PJG6.2	88.1	2.86
6	MCMBP	Q9BTE3.2	72.7	2.52
7	HELLS	Q9NRZ9.1	81.6	2.46

Table S2: Potential interaction partners of MYSM1 identified by mass spectrometry in 293T cells upon DNA damage induction by etoposide. Co-immunoprecipitated protein samples were subjected to SCX-HPLC-MS/MS and spectra were analyzed by MaxQuant Software. Abundance threshold: 4-16-fold (log2 = 4) as indicated.

Suppl. Figure 1

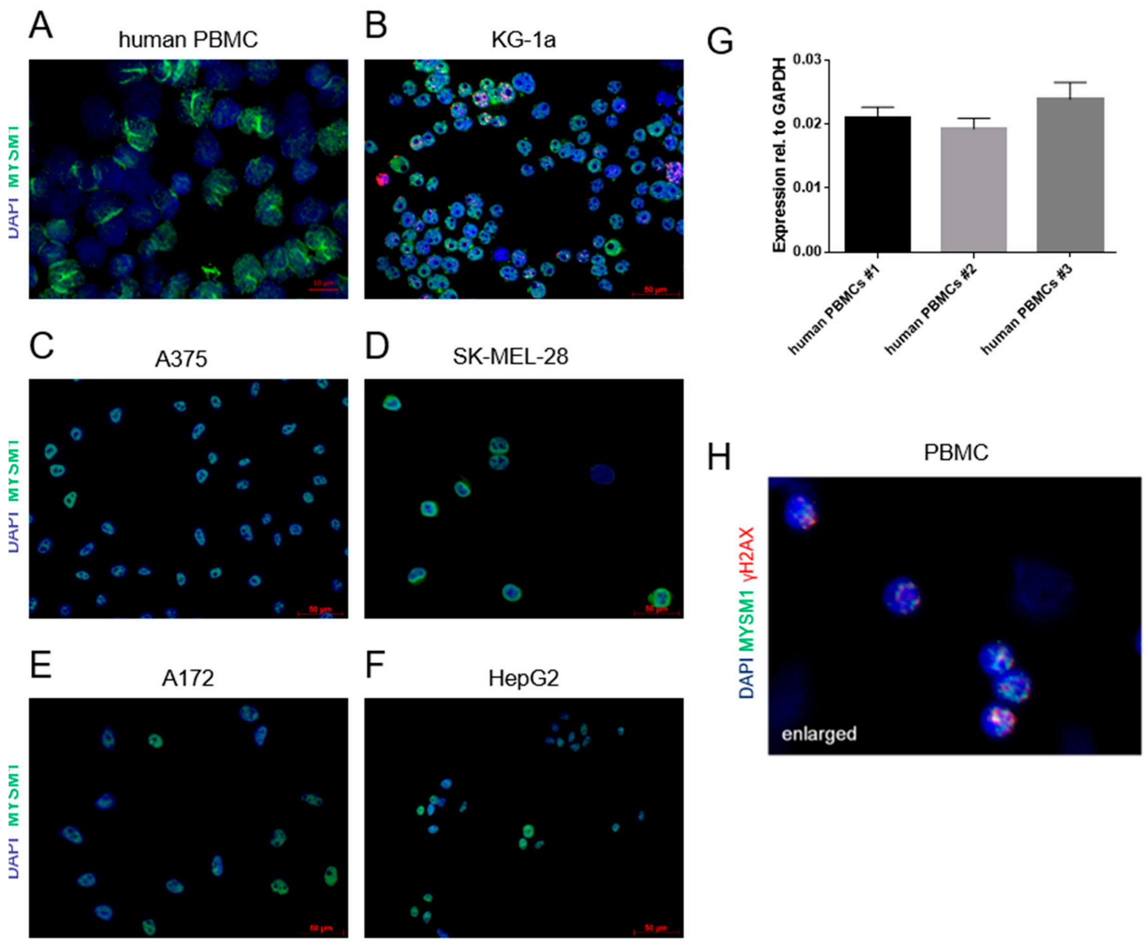

Figure 1. Expression of MYSM1 in human PBMC and indicated tumor cells lines. **A**.-**F**. IF analyses of MYSM1 expression and localization in human PBMC and indicated tumor cell lines (MYSM1 green, nuclei DAPI blue, red scale bars added for comparison, original magnification 20X or 40X) in (**A**). PBMC (confocal image) (**B**). KG-1a myeloid leukemia cells (**C**). A375 melanoma cells (**D**). SK-MEL-28 melanoma cells (**E**). A172 glioma cells (**F**). HepG2 liver cancer cells and (**G**). mRNA expression of MYSM1 in PBMC from three different donors and in indicated leukemia and lymphoma cell lines. (**H**). Colocalization of MYSM1 and γH2AX after treatment of human PBMC with 20 μM Etoposide for 16 hrs (MYSM1 green, γH2AX red, double-positive foci yellow).

Suppl. Figure 2

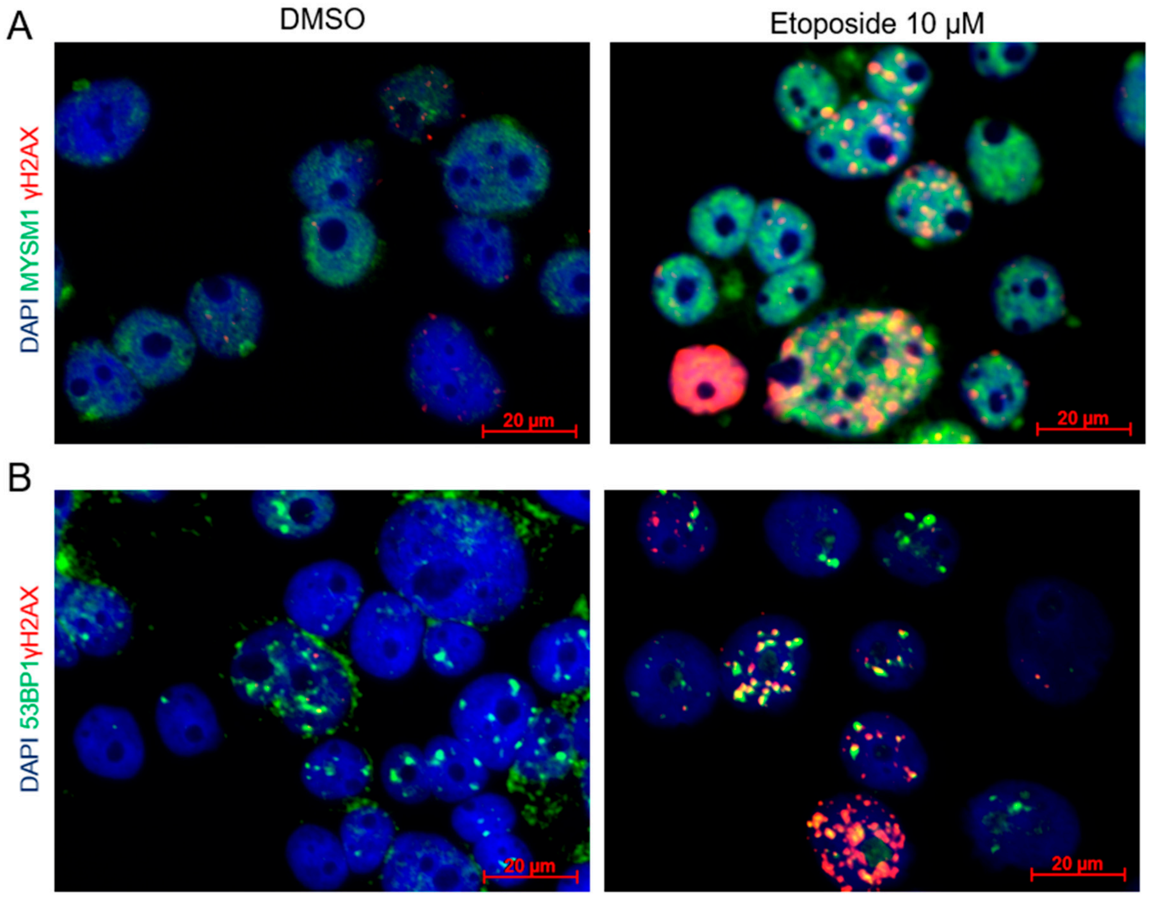

Figure 2. Induction of DNA damage responses upon etoposide treatment in KG-1a leukemia cells. IF analyses of γH2AX foci (red) and nuclei (blue) and colocalization with (**A**). MYSM1 (green) or (**B**). DNA damage marker 53BP1 (original magnification 63X, representative images of three independent experiments).

Suppl. Figure 3

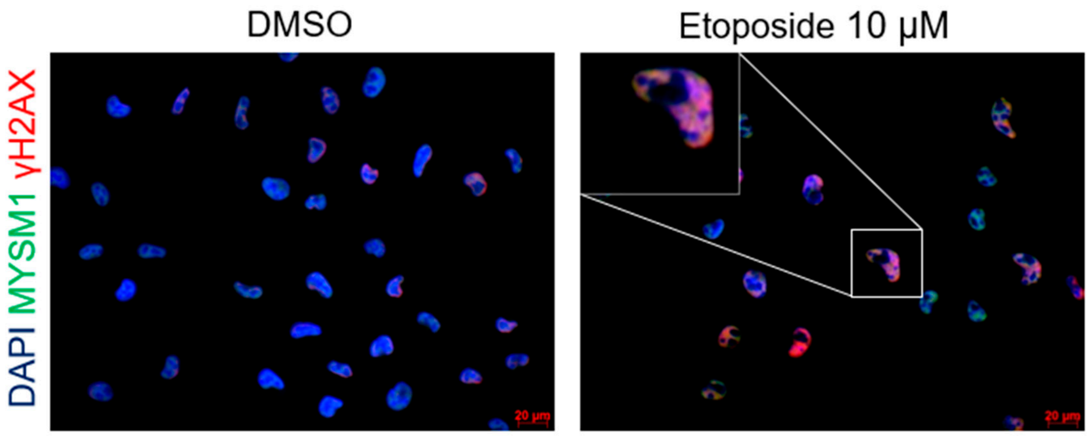

Figure 3. Recruitment of MYSM1 to γH2AX foci upon DSB induction with etoposide in A375 melanoma cells. IF analysis of γH2AX foci (red) formation and colocalization with MYSM1 (green) in the nuclei (blue) of human A375 melanoma cells treated with either DMSO or etoposide (10 µM) for 3 hrs as indicated (respresentative images, scale bars in red, original magnification 40X). A375 appeared to be highly sensitive to DNA damage as indicated by intense overall nuclear γH2AX staining. The insert shows an enlargement of representative A375 cells.

Suppl. Fig 4

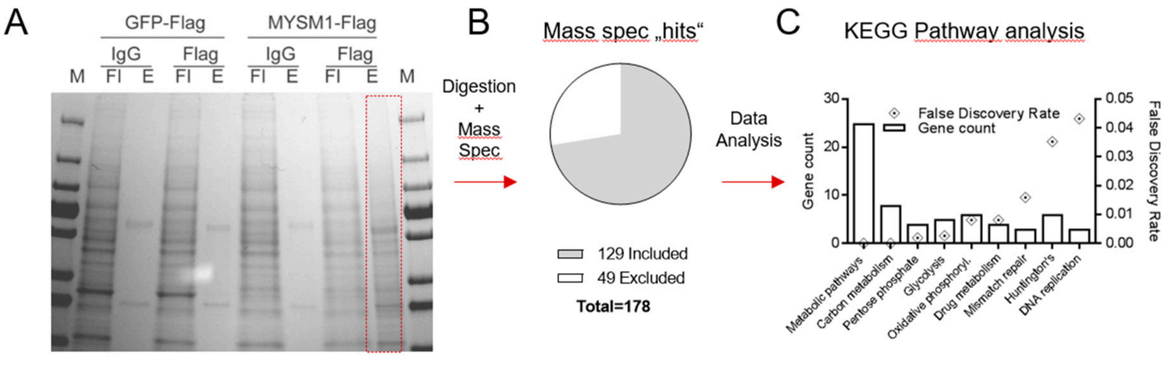

Figure 4. Procedure of the mass spectrometry analysis of eluates from Flag-IP samples. (**A**). Gel analysis of flow (Fl) and eluates (E) from anti-Flag IP samples of 293 T cell lysates prepared after transfection with either GFP-Flag and MYSM1-Flag constructs and subsequent treatment with etoposide or DMSO and prior to digestion as described in the Materials section. (**B**). Graph of overall number of MYSM1-Flag interacting peptids („hits") identified by mass spectrometry analysis. (**C**). Bar graph of pathway distribution of identified hits as per KEGG pathway analysis.

Suppl. Figure 5

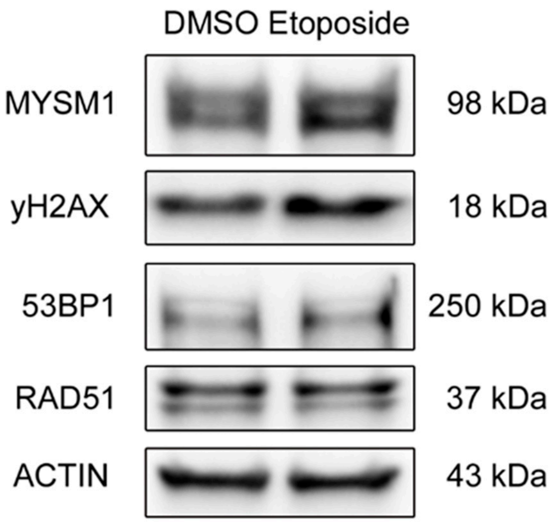

Figure 5. Western blot analysis of DNA damage marker expression in 293T cells upon exposure to etoposide. Lysates from 293T cells transfected with MYSM1-Flag and treated with etoposide or DMSO were prepared as described in the Materials section and analyzed by WB with antibodies against indicated DNA damage markers using ACTIN as loading control.

Suppl. Fig 6

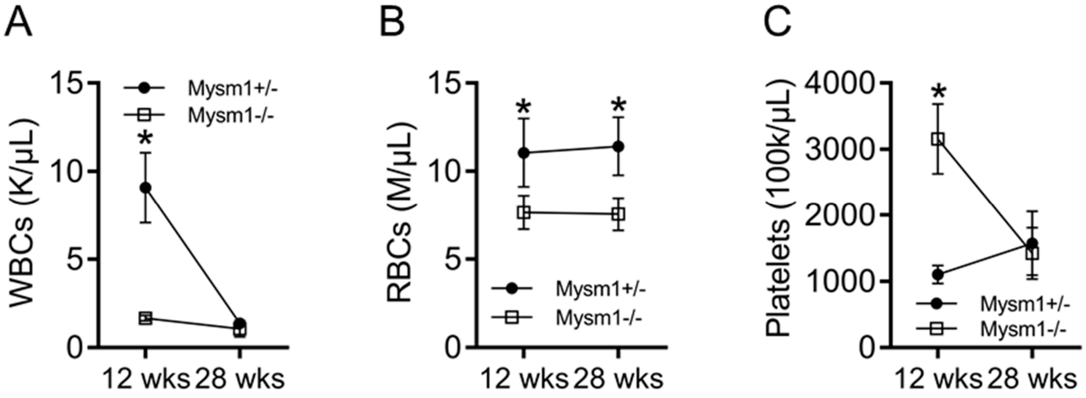

Figure 6. Blood cell counts and subsets in young vs. aged Mysm1-deficient mice and their Mysm1$^{+/-}$ littermates. (**A**). Leukocytes (**B**). Erythrocytes and (**C**). Thrombocytes.

Suppl. Figure 7

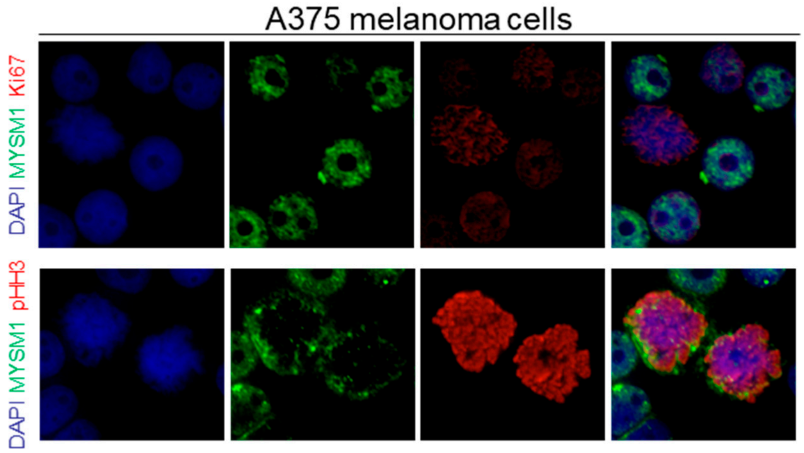

Figure 7. Cell-cycle dependent distribution of MYSM1 in A375 melanoma cells. **Upper panel**: IF analyses of Ki-67 foci (red) and colocalization with MYSM1 (green). **Lower panel**: IF analyses of Histon H3 (pHH3, red) and MYSM1 (green) in the nuclei (blue) of cycling A375 melanoma cells (original magnification 63X).

© 2020 by the authors. Licensee MDPI, Basel, Switzerland. This article is an open access article distributed under the terms and conditions of the Creative Commons Attribution (CC BY) license (http://creativecommons.org/licenses/by/4.0/).
